# Evaluation of the Idylla ctEGFR mutation assay to detect *EGFR* mutations in plasma from patients with non-small cell lung cancers

**DOI:** 10.1038/s41598-021-90091-z

**Published:** 2021-05-18

**Authors:** Pauline Gilson, Chloé Saurel, Julia Salleron, Marie Husson, Jessica Demange, Jean-Louis Merlin, Alexandre Harlé

**Affiliations:** 1 Service de Biologie Moléculaire des Tumeurs, Institut de Cancérologie de Lorraine, CNRS UMR 7039 CRAN-Université de Lorraine, 6 avenue de Bourgogne, CS 30519, 54519 Vandœuvre-lès-Nancy Cedex, France; 2 Département de Biopathologie, Institut de Cancérologie de Lorraine, 6 avenue de Bourgogne CS 30519, 54519 Vandœuvre-lès-Nancy Cedex, France; 3 Department of Biostatistics and Data Management, Institut de Cancérologie de Lorraine, 6 avenue de Bourgogne CS 30519, 54519 Vandœuvre-lès-Nancy Cedex, France

**Keywords:** Non-small-cell lung cancer, Cancer genomics

## Abstract

The assessment of *EGFR* mutations is recommended for the management of patients with non-small cell lung cancer (NSCLC). Presence of *EGFR* mutation is associated with response or resistance to EGFR tyrosine kinase inhibitors (EGFR-TKI). Liquid biopsy is nowadays widely used for the detection of resistance to EGFR-TKI. We evaluated here the performance of the Idylla ctEGFR mutation assay for the detection of *EGFR* mutations in circulating tumour DNA (ctDNA) in plasma from patients with NSCLC. Previously characterized plasma samples from 38 patients with NSCLC were analysed using 2 different analytical conditions (C1 and C2). The limit of detection (LOD) was evaluated using 2 mL of healthy donor plasma spiked with commercial DNA controls. Overall agreement, sensitivity and specificity were 92.1%, 86.7% and 95.7% for C1 condition respectively and 94.7%, 86.7% and 100% for C2 condition respectively. The T790M secondary resistance mutation was detected in two samples out of 3. The Idylla system was able to detect the exon 19 deletion from 6 copies/mL and up to 91 copies/mL for the G719S mutation. These results support that the Idylla ctEGFR mutation assay is a rapid option for the detection of *EGFR* hotspots mutations in plasma samples, however a particular attention is needed for its interpretation.

## Introduction

Lung cancer represents one of the most diagnosed cancers and the leading cause of death worldwide, contributing for 18.4% of all cancer deaths^1^. Non-small cell lung cancers (NSCLC) are the major histological subtype of lung neoplasms accounting for almost 85% of all cases^2^. Almost half of NSCLC patients are initially diagnosed with metastatic disease^3^ associated with limited therapeutic options and a dismal 5-year survival rate of ~ 2.9%^4^. Given the high prevalence and poor prognosis of NSCLC, considerable efforts have been made in the last decade to identify novel and more effective therapeutic strategies.

Better understandings of molecular mechanisms involved in NSCLC allowed the emergence of targeted therapies for NSCLC patients. *EGFR* (Epidermal growth factor receptor) was revealed as one of the major actionable oncogenic drivers in NSCLC development. Around 10% of NSCLC harboured *EGFR* activating mutations, predominantly in women, never- or light-smokers, adenocarcinomas and East Asian patients^5,6^. Hotspots *EGFR* mutations primarily occur between exons 18 and 21 in the EGFR tyrosine kinase coding domain^7,8^, leading to the upregulation of the downstream MAPK and PI3K/Akt/mTOR pro-oncogenic signaling pathways associated with excessive cell proliferation, survival, motility and invasion^9^. In-frame deletions in exon 19 and L858R single-point mutation in exon 21 are the most prevalent *EGFR* mutations, accounting for 45% and 40% of cases respectively^8^.

In clinical practice, patients with *EGFR-*mutated tumour and treated with first- to third-generation EGFR-TKI showed a better progression-free survival (PFS) compared to conventional chemotherapy, making EGFR-TKI in monotherapy the current standard of care for patients with advanced or metastatic *EGFR*-mutated NSCLC^10,11^. Despite an initial response to targeted therapies, most NSCLC with EGFR-TKI sensitizing mutations relapsed after 8–16 months of treatment due to the emergence of acquired drug resistance mechanisms^12^. The acquisition of a second-site *EGFR* T790M mutation in exon 20 was reported in more than half of NSCLC resistance cases^12–15^, conferring resistance to first- and second-generation EGFR-TKI and sensitivity to the sole third-generation inhibitor^16^.

Currently, *EGFR* mutation testing is considered as a prerequisite for the management of patients with advanced or metastatic NSCLC as it greatly helps for selecting patients for EGFR-targeted therapies, clonal evolution and resistance to treatment monitoring. Tumour tissue biopsy is commonly the gold-standard for molecular investigations in NSCLC. However, its use in clinical practice is limited by the lack of tissue material amenable to biopsy, the clonal heterogeneity of the tumour and the risk of surgical complications resulting from multiple biopsy procedures^17^. Recently, the analysis of circulating tumour DNA (ctDNA) shed into body fluids by tumour cells have emerged as a non-invasive and easily repeatable alternative to tissue biopsy^18^. Liquid biopsies based on ctDNA detection in plasma samples have now entered clinical routine for patients with NSCLC that cannot undergo tumour biopsy, whose biopsy gave non-contributive results or for monitoring. CtDNA analysis requires highly sensitive approaches given their fragmented nature and their dilution by cell-free DNA (cfDNA) shed by non-malignant cells in plasma making ctDNA only a low fraction (as low as 0.01%) of total cfDNA^19^.

The aim of this retrospective study was to evaluate the performance of the novel fully-automated PCR-based Idylla ctEGFR system for the detection of *EGFR* hotspot mutations in clinical plasma specimens from NSCLC patients and in laboratory-made spiked samples.

## Results

### Analysis of blood samples from NSCLC patients

For all 38 samples, both condition 1 (C1—no proteinase K incubation) and condition 2 (C2—30 min incubation) of the Idylla ctEGFR mutation assay were successful in generating valid results (Table 1). Based on the detection of EGFR-TKI sensitizing mutations, the Kappa coefficient of the two conditions was 0.83, 95% CI [0.64; 100], indicating a good overall agreement. Moreover, 35 out of the 38 samples (92.1%) tested with the C1 condition provided concordant results with the standard reference methods of the lab (Cobas EGFR mutation assay v2 and NGS assays). Among the three discrepant results, two samples were shown *EGFR* wild-type by Idylla C1 condition while found *EGFR*-mutated by Cobas EGFR mutation assay (L858R mutation in the #3 sample, G719X and S768I mutations in the #12 sample). Finally, the #22 sample harboured an exon 19 deletion by Idylla C1 condition while found *EGFR* wild-type by Cobas. Concerning EGFR-TKI sensitizing mutations, the sensitivity and specificity of the Idylla C1 condition were 86.7% and 95.7% respectively, using NGS or Cobas EGFR mutation v2 assay as the standard reference methods (Table 2).

**Table 1:** *EGFR* mutational status obtained by a standard reference method (custom capture-based NGS or Cobas EGFR mutation assay v2) and the Idylla ctEGFR mutation assay (C1 and C2 conditions).

Sample ID	Standard reference method		Idylla ctEGFR mutation assay							
			C1 condition				C2 condition			
	Technique	Mutation	Genetic call	Mutant Cq	SPC Cq	∆Cq	Genetic call	Mutant Cq	SPC Cq	∆Cq
1	Cobas	L861Q	L861Q	33.0	22.6	10.5	L861Q	32.2	22.3	10.0
2	Cobas	L858R	L858R	26.1	22.0	4.1	L858R	26.0	22.1	3.9
3	Cobas	L858R	**WT**	–	21.2	–	L858R	30.2	21.1	8.9
4	Cobas	L858R	L858R	27.7	19.6	8.1	L858R	26.6	19.3	7.5
5	NGS	L858R	L858R	27.4	19.6	7.7	L858R	26.7	19.1	7.5
6	Cobas	L858R	L858R	26.1	21.6	4.2	L858R	25.7	21.8	3.7
7	Cobas	DEL19	DEL19	30.1	18.4	11.6	DEL19	30.0	18.1	11.7
8	Cobas	DEL19	DEL19	24.9	19.7	5.2	DEL19	24.7	19.1	5.4
9	Cobas	DEL19	DEL19	26.1	21.5	4.9	DEL19	26.9	21.3	5.8
10	Cobas	DEL19	DEL19	34.1	22.3	11.6	**WT**	–	22.1	-
11	Cobas	DEL19	DEL19	29.5	21.5	8.0	DEL19	26.8	20.6	6.3
12	Cobas	G719X	**WT**	–	20.0	–	**WT**	–	19.7	–
		S768I	**WT**	–	20.0	**–**	**WT**	–	19.7	–
13	Cobas	DEL19	DEL19	26.4	19.9	6.3	DEL19	27.1	20.5	6.6
		T790M	T790M	25.0	19.9	5.2	T790M	25.8	20.5	5.7
14	Cobas	DEL19	DEL19	24.3	20.7	3.4	DEL19	24.4	20.9	3.4
		T790M	T790M	25.4	20.7	5.0	T790M	25.5	20.9	5.0
15	Cobas	DEL19	**DEL19**	32.0	22.7	9.0	**DEL19**	32.7	23.2	9,5
		T790M	**WT**	–	22.7		**WT**	–	23.2	–
16	Cobas	WT	WT	–	21.0	–	WT	–	21.8	–
17	Cobas	WT	WT	–	22.5	–	WT	–	22.6	–
18	Cobas	WT	WT	–	22.4	–	WT	–	22.6	–
19	Cobas	WT	WT	–	22.2	–	WT	–	22.4	–
20	Cobas	WT	WT	–	23.2	–	WT	–	23.6	–
21	Cobas	WT	WT	–	21.6	–	WT	–	21.5	–
22	Cobas	WT	**DEL19**	35.0	22.1	12.6	WT	-	22.7	–
23	Cobas	WT	WT	–	21.7	–	WT	–	21.7	–
24	Cobas	WT	WT	–	22.3	–	WT	–	22.8	–
25	Cobas	WT	WT	–	20.5	–	WT	–	20.5	–
26	NGS	WT	WT	–	21.5	–	WT	–	21.7	–
27	Cobas	WT	WT	–	19.8	–	WT	–	19.9	–
28	Cobas	WT	WT	–	20.3	–	WT	–	20.7	–
29	Cobas	WT	WT	–	21.9	–	WT	–	20.9	–
30	Cobas	WT	WT	–	20.9	–	WT	–	21.2	–
31	Cobas	WT	WT	–	18.4	–	WT	–	18.5	–
32	Cobas	WT	WT	–	19.6	–	WT	–	20.0	–
33	NGS	WT	WT	–	22.0	–	WT	–	21.7	–
34	Cobas	WT	WT	–	19.4	–	WT	–	20.1	–
35	Cobas	WT	WT	–	21.0	–	WT	–	20.8	–
36	Cobas	WT	WT	–	15.7	–	WT	–	15.6	–
37	Cobas	WT	WT	–	23.2	–	WT	–	23.5	–
38	NGS	WT	WT	–	22.4	–	WT	–	22.3	–

The discrepant results between the Idylla assay (C1 or C2) and the reference method are mentioned in bold.

**∆**Cq was defined as the difference between mutant (mutant Cq) and internal control (SPC Cq) signals.

*Cq* cycle of quantification, *DEL19* exon 19 deletion, *SPC* sample processing control, *WT* wild-type.

**Table 2:** Overall agreement, sensitivity and specificity of the Idylla ctEGFR mutation assay (C1 and C2 conditions) for the detection of EGFR-TKI sensitizing mutations.

	Idylla C1 condition	Idylla C2 condition
Overall agreement with the reference methods^a^	92.1% [83.5%; 100%] (35/38)	94.7% [87.6%; 100%] (36/38)
Sensitivity^a^	86.7% [69.5%; 100%] (13/15)	86.7% [69.5%; 100] (13/15)
Specificity^a^	95.7% [87.3%;100%] (22/23)	100% (23/23)

^a^Overall agreement, sensitivity and specificity of each condition is expressed as a percentage with 95% confidence interval (95% CI) and the number of samples used to calculate them. The NGS and Cobas EGFR mutation assays were set as the gold standard.

The C2 condition of the Idylla assay demonstrated agreement with the standard reference methods in 36 of the 38 cases for the detection of EGFR-TKI sensitizing mutations (94.7%) (Table 1). A total of 2 discordant results were observed between the Idylla C2 condition and the Cobas assays: the #10 and #12 samples were found *EGFR* wild-type by the Idylla C2 condition while having an exon 19 deletion and G719X/S768I genotypes respectively by Cobas. Using standard approaches of the lab as the reference for the detection of EGFR-TKI sensitizing mutations, the C2 condition of the Idylla assay reached a 86.7% sensitivity and a 100% specificity (Table 2).

Concerning the three samples found with a secondary resistance mutation (#13–#15) by a standard reference method, both conditions of the Idylla system detected the T790M mutation in two cases (#13–#14) and missed it in one case (#15) (Table 1).

### Limit of detection (LOD) determination using laboratory-made samples

Different volumes of the commercial DNA solutions were added into 2 mL of commercial plasma in order to obtain expected mutant allele frequencies (MAF) that range from 0.015% (1 mutant copy/6843 cfDNA copies, 2.6 copies/mL) to 0.714% (1 mutant copy/140 cfDNA copies, 122 copies/mL). All dilutions were detailed in Supplementary Table S1 online and representative fragment length profiles of the DNA extracted from these samples were reported in Supplementary Fig. S1 online. All artificial specimens gave contributive results with the C1 and C2 conditions of the Idylla system. Commercial plasma alone and plasma spiked with *EGFR* wild-type DNA solution served as controls and gave “no mutation” results by Idylla C1 and C2 conditions (Table 3). Using the Idylla C1 condition, the LOD were 0.556% (91 copies/mL) for the G719S mutation, 0.144% (25 copies/mL) for the S768I mutation, 0.112% (18 copies/mL) for the L861Q and V769_D770ins mutations, 0.063% (10 copies/mL) for the L858R and T790M mutations and 0.041% (6 copies/mL) for the E746_A750del mutation (Table 3). The LOD obtained by Idylla C2 condition were 0.556% (91 copies/mL) for the G719S mutation, 0.144% (25 copies/mL) for the L861Q, T790M and V769_D770ins mutations, 0.112% (18 copies/mL) for the L858R mutation and 0.063% (10 copies/mL) for the S768I and the E746_A750del mutations.

**Table 3:** Limits of detection (LOD) of the Idylla ctEGFR mutation assay for the detection of 7 *EGFR* hotspots mutations using C1 or C2 condition.

*EGFR* variant	Ratio mutant copies/total cfDNA copies (%)	C1 condition			C2 condition		
		Mutant Cq	SPC Cq	Biological interpretation	Mutant Cq	SPC Cq	Biological interpretation
L861Q	0.455	30.0	6.4	Detected	30.8	7.9	Detected
	0.144	31.8	10.4	Detected	32.7	10.4	**Detected**
	0.112	33.5	10.3	**Detected**	–	–	Not detected
	0.092	–	–	Not detected	–	–	–
L858R	0.112	30.0	6.4	Detected	28.2	4.6	**Detected**
	0.092	29.9	6.8	Detected	–	–	Not detected
	0.063	30.0	6.5	**Detected**	–	–	–
	0.041	–	–	Not detected	–	–	–
S768I	0.144	35.8	13.9	**Detected**	36.3	14.0	Detected
	0.112	–	–	Not detected	38.3	15.0	Detected
	0.092	–	–	–	36.9	12.9	Detected
	0.063	–	–	–	36.3	12.7	**Detected**
	0.041	–	–	–	–	–	Not detected
V769_D770ins	0.144	32.5	10.4	Detected	32.5	9.7	**Detected**
	0.112	35.0	11.4	**Detected**	–	–	Not detected
	0.092	–	–	Not detected	–	–	–
T790M	0.144	29.4	7.9	Detected	29.7	7.3	**Detected**
	0.112	31.9	9.2	Detected	–	–	Not detected
	0.092	30.6	7.8	Detected	–	–	–
	0.063	32.4	9.5	**Detected**	–	–	–
	0.041	–	–	Not detected	–	–	–
E746_A750del	0.063	35.6	12.3	Detected	33.4	9.7	**Detected**
	0.041	37.2	13.0	**Detected**	–	–	Not detected
	0.033	–	–	Not detected	–	–	–
G719S	0.714	35.9	13.7	Detected	34.9	13.1	Detected
	0.556	35.1	12.2	**Detected**	37.4	14.4	**Detected**
	0.455	–	–	Not detected	–	–	Not detected
EGFR wild-type	0	–	–	Not detected	–	–	Not detected
Commercial plasma	–	–	–	Not detected	–	–	Not detected

For the determination of the LOD, artificial plasma samples were extemporaneously prepared in the lab by spiking *EGFR* multiplex commercial cfDNA controls in plasma from healthy donors. Details on the preparation of spiked plasma samples are detailed in Supplementary Table S1. Limits of detection are mentioned in bold.

## Discussion

Plasma *EGFR* genotyping is now commonplace in clinical care for patients with newly diagnosed NSCLC or NSCLC progressive disease^20^. Liquid biopsies offer a non-invasive surrogate to tissue biopsy, particularly in patients with NSCLC where tumour tissue is sometimes scarce or difficult to sample.

Here, we tested the qPCR-based Idylla ctEGFR mutation assay for the detection of *EGFR* mutations in cfDNA. The LOD for 7 *EGFR* hotspot mutations was determined using artificial samples containing different levels of spiked cfDNA in healthy donor plasma. Two conditions of the Idylla ctEGFR mutation assay were evaluated. Both C1 and C2 conditions comprised the addition of proteinase K at room temperature to digest plasma proteins and remove protein-DNA bounds^21^. The C2 condition included a supplementary 30 min incubation to assess if changing proteinase K conditions could affect the error rate of the Idylla system or its performance to detect mutations. Both conditions tested finally gave valid results for all the artificial samples analysed. Using the C1 condition, the LOD ranged from 6 copies/mL (0.041%) for the exon 19 deletion to 91 copies/mL (0.556%) for the G719S mutation. The LOD of the Idylla approach using the C2 condition were from 10 copies/mL (0.063%) for the S768I mutation and the exon 19 deletion to 91 copies/mL (0.556%) for the G719S mutation. Except for the S768I mutation, the C1 condition seems to provide lower LOD than the C2 condition, however this difference need to be confirmed by further analyses.

It should be noticed that the LOD for each mutation was defined in our study as the lowest allele frequency for which a “mutation call” is reported before obtaining "no mutation detected" results for lower MAF. In very few cases, MAF lower than the LOD yielded to the detection of an *EGFR* mutation probably due to stochastic variance (in one run, sufficient mutant copies end up in the PCR chamber and lead to exponential amplification while in the following runs, a “no mutation call” will be obtained). This phenomenon is not reproducible and therefore has not be taken into account for the LOD estimation.

Among the other techniques available for *EGFR* mutation detection in cfDNA^18^, the commercial Cobas EGFR mutation assay v2 kit is one of the most quantitative PCR methods investigated and the only FDA-approved companion diagnostic for the detection of exon 19 deletion, L858R and T790M mutations in plasma samples from NSCLC patients^22^. The thresholds for calling *EGFR* plasma mutations by Cobas method were in the same range than those described in our study for the Idylla assay: 5–27 copies/mL (0.10–0.51%) for the exon 19 deletion, 35–70 copies/mL (0.39–0.80%) for the L858R mutation and 18–36 copies/mL (0.38–0.81%) for the T790M mutation^23^. Commercial or custom digital PCR approaches reach better sensitivities than the Idylla approach and seem more appropriate for the detection of very low mutant allele fractions. These include the droplet digital PCR (ddPCR) method with an estimated LOD of 0.05% for the exon 19 deletion and L858R mutations and 0.1% for T790M mutations^24^ and the OncoBEAM (beads, emulsion, amplification and magnetics) approach with a LOD of nearly 0.02% for T790M mutation^25^. The conventional NGS-based approaches are considered less sensitive, however the implementation of a molecular tagging strategies in NGS technology in the last years allows to significantly reduce the sequencing artifacts and reach sensitivities similar to those observed with the other approaches (up to 0.1%)^26,27^.

The Idylla approach interrogates a limited number of EGFR-TKI sensitizing and resistance mutations with an *EGFR* panel quite similar to that covered by the Cobas approach. Both Idylla and Cobas methods are able to detect the *EGFR* T790M mutation conferring a resistance to the first- and second-generation EGFR-TKI but are not originally designed for the research of the *EGFR* C797S resistance mutation to the third-generation EGFR-TKI. Conversely, NGS represents a broader approach that covers non-hotspot regions of the *EGFR* gene as well as mutations in other cancer-related genes which could be targeted or reveal specific mechanisms of resistance^18^.

Among the different approaches investigated, the Idylla and Cobas methods account for the fastest approaches with total turnaround times that do not exceed 160 min and 3.5 h respectively. Moreover, the Idylla system is the solely automated approach that integrates the extraction process and only requires 5 min hands-on-time. By contrast, the NGS approaches have prolonged turnaround times (2.5–4 days depending on the strategy and the enrichment method used) compared to the previous methods.

Finally, regarding the cost of the reagents and equipment needed for the different approaches, Idylla and Cobas are the most attractive methods (< 250 euros/sample) compared to the commercial or custom NGS approaches (> 300 euros/sample).

The clinical performance of the Idylla ctEGFR mutation assay was also evaluated by analyzing different NSCLC specimens previously genotyped by the standard testing methods in our lab (a custom capture-based NGS approach and the Cobas EGFR mutation assay v2). Both C1 and C2 conditions of the Idylla ctEGFR mutation assay led to valid results and demonstrated a good overall concordance (κ = 0.83) for the determination of EGFR-TKI sensitizing mutations. Considering only the EGFR-TKI sensitizing mutations, the Idylla ctEGFR mutation assay (whatever the condition used) reached high concordance, sensitivity and specificity compared to standard reference methods. Given the confidence intervals calculated, we could not determine which condition gave better results. The discordances observed for samples #3 and #10 seem to likely result from negative results given that, in both cases, one out of the two conditions retrieved the mutation identified by Cobas. With regard to sample #12, the Cobas assay detected 2 EGFR-TKI sensitizing mutations (G719X and S786I) while the Idylla system resulted in “no mutation call” in both conditions. Subsequently, a tissue biopsy was available for NGS analysis and retrieved the same mutations (G719C with MAF = 4.9% and S768I with MAF = 6.6%) than those obtained by Cobas. As both mutations were found with Cobas and NGS, it is expected that the mutations were present in the samples although in too low allele frequencies in the plasma to be detected with the Idylla ctEGFR mutation assay. For sample #22, it was difficult to point out the cause of the discordance. The exon 19 deletion identified by the Idylla C1 condition was a borderline positive call (Cq = 35, **∆**Cq = 12.6) whereas Cobas and Idylla C2 condition did not find any *EGFR* mutation. Based on this data, we could assume that the deletion identified by the Idylla C1 condition was a false-positive result due to the low amount of amplifiable DNA (SPC Cq of 22.1 and 22.7 for C1 and C2 conditions respectively) that led to a background signal which was faulty called positive. However, an exhaustive analysis of the amplification curves obtained by the Idylla C2 condition led to the identification of a non-valid amplification curve for an exon 19 deletion. Thus, we could also consider that the Cobas could have missed this variant if not included in the panel covered or present with very low frequency.

For cases with a secondary resistance mutation, the Idylla system succeeded in retrieving the T790M mutation obtained by the reference method in two out of the three clinical samples tested (#13–#14) and missed it in one case (#15). In this latter case however, a thorough analysis of the amplification curves was performed and the T790M mutation was identified in the C1 and C2 conditions of the Idylla assay as a borderline negative result filtered by the software during the analysis process. The internal controls present in the cartridge (SPC Cq of 22.7 and 23.2 for C1 and C2 conditions respectively) indicated a rather low amount of amplifiable DNA that could explain the false-negative results.

These data should be examined in the light of some limitations. First, due to the limited number of clinical samples available, only the most common clinically significant *EGFR* mutations covered by the Idylla panel have been tested and the exact reason of some discordances could not be identified. Second, the Idylla system requires smaller volumes of plasma (2 mL) to those used in routine practice for NGS and Cobas approaches (4 mL), thus less ctDNA copies are present in the sample analysed, which could explain some of the false-negative results obtained by Idylla.

To our knowledge, no study which evaluated the clinical performance of the Idylla ctEGFR mutation assay in plasma samples were published at the moment. However, the LOD of the Idylla ctDNA KRAS and NRAS/BRAF mutation assays have been already evaluated in a previous work and were found in the same range than those observed in our study^21^. Moreover, numerous studies have already reported the performance of the Idylla EGFR mutation assay for the detection of *EGFR* hotspot mutations using tumour tissue sections^28–34^ or DNA extracted from tissue samples^35,36^. The Idylla EGFR mutation assay had the advantage to retrieve samples that did not reach DNA quality requirements for NGS analysis^37^. However, some studies reported a lack of sensitivity of the system, particularly for the detection of the *EGFR* T790M mutation^38,39^. Some false negative results could be explained by the presence of the Q787Q polymorphism that interferes with the detection of the *EGFR* T790M mutation nearby^38^. Considering the high specificity and moderate sensitivity of the Idylla EGFR system, some groups recognize this assay as a suitable rapid first screen assay and suggest NGS as a mandatory complementary analysis for samples with no EGFR mutation detected using Idylla or for samples with low amounts of tumour material/DNA^39–42^. Here, we showed that the Idylla ctEGFR mutation assay seems to exhibit the same advantages and caveats than the previously described tissue-based Idylla EGFR mutation assay. In the same manner, an integrative workflow including a first analysis by Idylla ctEGFR system followed by a comprehensive genome profiling by NGS should also be considered if sufficient plasma is available.

To conclude, the Idylla system seems to be easily implemented into all clinical laboratories and meets the urgent need for clinicians to guide treatment in function of tumour evolution. The Idylla ctEGFR mutation assay reaches a high specificity and could be proposed as a first screen to detect *EGFR* hotspots mutations. However, due to false negative results, a careful interpretation is needed for cases with low amounts of amplifiable cfDNA or for the particular research of the T790M mutation in the context of *EGFR*-mutated NSCLC progressing after treatment. Thorough analysis of amplification curves obtained by Idylla is needed. Moreover, considering the crucial stakes of *EGFR* status in the management of NSCLC patients at diagnosis and during disease monitoring, orthogonal assays should be systematically performed in cases of low DNA amounts and EGFR WT results.

## Material and methods

### Study design and sample selection

**Figure 1:**
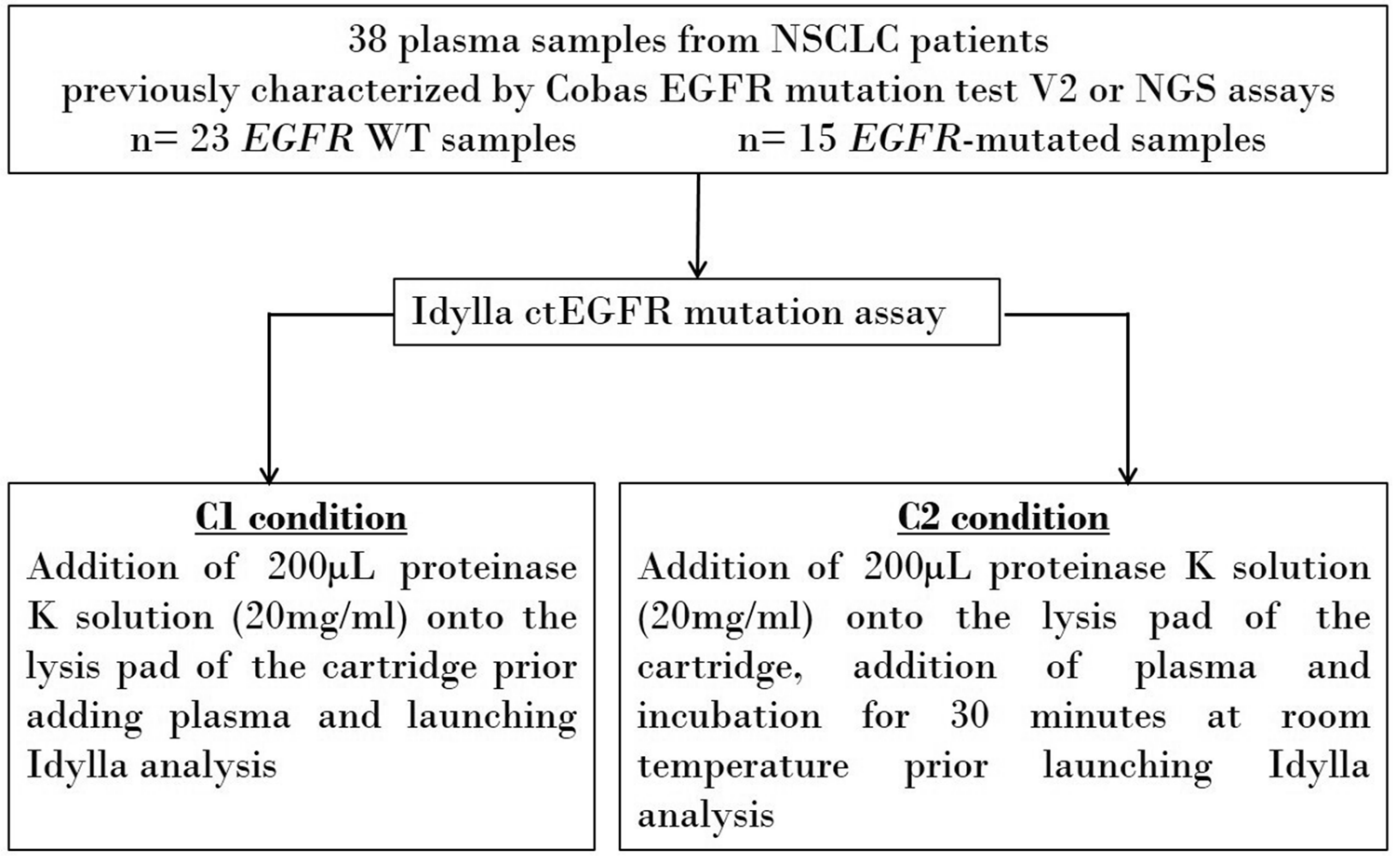
Workflow of the retrospective study on clinical plasma samples from NSCLC patients. *NGS* next-generation sequencing, *NSCLC* non-small cell lung cancers, *WT* wild-type.

A total of 38 blood samples from NSCLC patients were retrospectively chosen among the biological collection of Institut de Cancérologie de Lorraine (ICL, Vandœuvre-lès-Nancy, France). Blood samples were obtained during standard routine care for NSCLC patient cancer management that includes an *EGFR* genotyping by a lab reference method (Cobas EGFR mutation test V2 or NGS assays) (Fig. 1). All patients gave their written informed consent for the use of their biological samples for research purposes. The clinical characteristics of the patients are listed in Table 4. The study received approval from the ethical and scientific board of Institut de Cancérologie de Lorraine. All experiments were performed in accordance with the relevant guidelines and regulations. Data were anonymized in the time of patient inclusion. Interpretation of the Idylla results was performed by an experienced biologist who was blinded to the routine care results.

**Table 4:** Clinical characteristics of the 38 enrolled patients.

	All patients (N = 38)
**Sex**	
Female	24 (63.2%)
Male	14 (36.8%)
Age at diagnosis (years) median [interquartile range]	74 [62–84]
**Smoking status**	
Non-smoker	12 (31.6%)
Former smoker	8 (21.1%)
Current smoker	5 (13.2%)
Not provided	13 (34.2%)
**NSCLC histological subtype (according to 2015 WHO classification)**	
Adenocarcinoma	36 (94.7%)
Large cell carcinoma	2 (5.3%)
**NSCLC stage**	
Stage III	1 (2.6%)
Stage IV	37 (97.4%)
**Sampling time**	
At initial diagnosis	27 (71.1%)
At clinical progression under EGFR-TKI treatment	11 (28.9%)

*EGFR-TKI* EGFR tyrosine kinase inhibitors, *NSCLC* non-small Cell Lung cancer, *WHO* World Health Organization.

### Blood collection and initial sample processing

For each patient, blood samples were drawn into two 10 mL Streck Cell-Free DNA BCT collection tubes (Streck Inc., Omaha, NE, USA) and plasma processing was performed within 3 days after blood collection. Whole blood was first spun at 1600*g* for 10 min followed by a second centrifugation of the supernatant at 6000*g* for 10 min. Plasma were then isolated and stored at -80 °C before further analyses.

### CfDNA extraction and fragment length distribution analysis

For Cobas EGFR mutation assay, cfDNA was extracted from plasma using the Cobas cfDNA sample preparation kit (Roche Diagnostics, Meylan, France) following the manufacturer’s recommendations. For NGS analysis, cfDNA was extracted from plasma using the QIAamp Circulating Nucleic Acid Kit (Qiagen, Hilden, Germany) and quantified using Qubit 3.0 Fluorometer instrument (ThermoFisher Scientific, Courtaboeuf, France) and the Qubit dsDNA HS Assay Kit (Thermo Fisher Scientific). For both reference methods, the volume of plasma used for DNA extraction was variable, depending on the volume of blood collected and the yield of the initial blood processing (Table 5). No previous cfDNA extraction was required prior to Idylla analysis.

**Table 5:** Biological characteristics of the samples.

Sample ID	Volume of plasma used (mL)	Standard reference method used (details on DNA input for NGS analyses)	*EGFR* mutational status				
			Exon	Mutation	Base change	Amino acid change	Proportion of *EGFR-*mutated versus *EGFR* wild-type copies
1	4	Cobas	21	L861Q	c.2582T>A	p.(Leu861Gln)	2.61
2	4	Cobas	21	L858R	c.2573T>G; c.2573_2574delinsGT	p.(Leu858Arg)	10.07
3	4	Cobas	21	L858R	c.2573T>G; c.2573_2574delinsGT	p.(Leu858Arg)	6.41
4	4.5	Cobas	21	L858R	c.2573T>G; c.2573_2574delinsGT	p.(Leu858Arg)	6.07
5	4	NGS (138 ng)	21	L858R	c.2573T>G	p.(Leu858Arg)	VAF = 1.2% (9242 read depth)
6	3	Cobas	21	L858R	c.2573T>G; c.2573_2574delinsGT	p.(Leu858Arg)	10.40
7	3.5	Cobas	19	DEL19	deletion	–	13.45
8	4	Cobas	19	DEL19	deletion	–	16.80
9	4	Cobas	19	DEL19	deletion	–	15.30
10	4	Cobas	19	DEL19	deletion	–	9.62
11	5	Cobas	19	DEL19	deletion	–	11.01
12	4.5	Cobas	18	G719X	c.2156G>C; c.2155G>T; c.2155G>A	p.(Gly719Ala) ; p.(Gly719Cys) ; p.(Gly719Ser)	1.51
			20	S768I	c.2303G>T	p.(Ser768Ile)	3.48
13	4	Cobas	19	DEL19	deletion	–	17.26
			20	T790M	c.2369C>T	p.(Thr790Met)	12.37
14	4	Cobas	19	DEL19	deletion	–	16.78
			20	T790M	c.2369C>T	p.(Thr790Met)	11.27
15	4	Cobas	19	DEL19	deletion	–	12.25
			20	T790M	c.2369C>T	p.(Thr790Met)	5.98
16	4.5	Cobas	WT				–
17	4	Cobas	WT				–
18	4	Cobas	WT				–
19	4	Cobas	WT				–
20	4	Cobas	WT				–
21	4	Cobas	WT				–
22	4	Cobas	WT				–
23	4.5	Cobas	WT				–
24	4	Cobas	WT				–
25	4.3	Cobas	WT				–
26	4	NGS (27 ng)	WT				–
27	2.5	Cobas	WT				–
28	4.5	Cobas	WT				–
29	4.5	Cobas	WT				–
30	4.5	Cobas	WT				–
31	4	Cobas	WT				–
32	5	Cobas	WT				–
33	4.3	NGS (19 ng)	WT				–
34	5	Cobas	WT				–
35	4	Cobas	WT				–
36	4	Cobas	WT				–
37	5	Cobas	WT				–
38	5	NGS (23 ng)	WT				–

The Cobas EGFR mutation test v2 is designed to detect 42 *EGFR* mutations but does not distinguish allele variants as specific L858R mutations or specific exon 19 deletions. In the same manner, G719A, G719C and G719S mutations cannot be differentiated by Cobas and are all referred as G719X. The proportion of *EGFR*-mutated *versus EGFR* wild-type copies is estimated by the variant allele frequency (VAF) for NGS analysis or by a semiquantitative index (SQI) for Cobas analysis.

*DEL19* exon 19 deletion, *VAF* variant allele frequency, *WT* wild-type.

### Cobas EGFR mutation test v2

The Cobas EGFR mutation test v2 (Roche Diagnostics) is a real-time PCR assay for the qualitative detection of 42 hotspots mutations in exons 18–21 of the *EGFR* gene^43^. The Cobas EGFR mutation test v2 assay was performed following the manufacturer's indications. Analysis of each sample was performed in three separate assays. For each assay, the PCR reaction mixture was prepared using standard input of 25 µL of extracted cfDNA, 20 µL of master mix solution and 5 µL of magnesium acetate. Negative and positive controls included in the kit as well as a no-template control were systematically tested in each run. PCR amplification was performed on the Cobas z480 analyzer and results were treated using the cobas 4800 software. A semiquantitative index (SQI) was calculated for every *EGFR* mutation detected in cfDNA and estimates the proportion of *EGFR-*mutated versus *EGFR* wild-type copies^44^. This in-vitro diagnostic assay gives results in less than 4 h.

### Capture-based NGS

Library preparation was performed from extracted DNA using a custom 51-gene capture-based “Solid Tumor Solution” kit (Sophia Genetics, Saint-Sulpice, Switzerland) as previously described^21,45^. The DNA input ranges from 19 ng (#33) to 138 ng (#5) depending on the DNA yield during the extraction process (Table 5). The kit allows the sequencing of different regions of clinical interest in 51 cancer-associated genes (*AKT1, ALK, ARID1A, BRAF, BRCA 1, BRCA 2, CDK4, CDKN2A, CTNNB1, DDR2, DICER1, EGFR, ERBB2, ERBB4, ESR1, FBXW7, FGFR1, FGFR2, FGFR3, FOXL2, GNA11, GNAQ, GNAS, H3F3A, H3F3B, HIST1H3B, HRAS, IDH1, IDH2, KIT, KMT2A, KMT2D, KRAS, MAP2K1, MAP2K2, MET, MTOR, MYOD1, NRAS, PDGFRA, PIK3CA, PTPN11, RAC1, RAF1, RET, ROS1, SF3B1, SMAD4, TERT, TGFBR2* and *TP53* genes). Libraries were sequenced using the MiSeq platform (Illumina, San Diego, CA, USA) and NGS raw data were analysed using the Sophia DDM software version 5.5.0 (Sophia Genetics). The pipeline v5.5.11 used for the bioinformatics analysis was provided by Sophia Genetics and consists in an alignment tool for the generation of bam files from Fastq files (hg19 reference genome) and a variant caller for the determination of single-nucleotide variations and insertion-deletions. A minimum of 1500× depth and 95% coverage were required for each sample analysed by NGS. The limit of detection (LOD) of this approach was determined at 0.5% variant allele frequency for 6000× total read depth.

### Biological characteristics of the selected samples

Blood samples were collected between January 2019 and July 2020. An average of 4.1 mL of plasma (interquartile range [4; 4]) were used for DNA extraction (Table 5). Thirty-four out of the 38 samples were analysed by Cobas and 4 samples were analysed by capture-based NGS (#5, #26, #33, #38). Based on these two standard reference methods, 15 samples were defined as *EGFR*-mutated (#1–#15). Among them, 11 samples harboured a single *EGFR* mutation, including one sample with a L861Q mutation (#1), five samples with a L858R mutation (#2–6) and five samples with an exon 19 deletion (#7–#11). Four samples were defined as having two different *EGFR* mutations: one sample with G719X and S768I *EGFR* sensitizing mutations (#12) and three samples with an exon 19 deletion and a T790M resistance mutation (#13–#15). Finally, the last 23 samples were found *EGFR* wild-type.

### Idylla ctEGFR mutation assay

The Idylla ctEGFR mutation assay (Biocartis NV, Mechelen, Belgium) is a real-time PCR assay designed for the qualitative detection of 49 mutations of the *EGFR* gene in plasma samples (see Supplementary Table S2 online). The Idylla platform provides a fully-automated system based on the use of cartridges with all reagents on-board. In this study, 2 mL of plasma were used for each assay and two conditions were tested for each sample (Fig. 1).

- The C1 condition consisted in the addition of 200 µL of proteinase K (20 mg/mL) (reference 19133, Qiagen) into the lysis pad of the cartridge then the split addition of the 2 mL of plasma sample into the cartridge.
- The C2 condition resumed the previous protocol followed by an incubation in the cartridge for 30 min at room temperature.

For both conditions, the cartridge was finally sealed and inserted into the instrument to launch the analysis. Inside the single-use cartridge, DNA was prepared and amplified by real-time PCR then detected using a fluorophore-based system. PCR amplification curves were analysed by the Idylla console software and cycle of quantification (Cq) values were determined. Sample Processing Control (SPC) signal corresponded to the amplification of *EGFR* wild-type internal control and ensured that DNA was present in sufficient quantity and quality for the analysis. If the difference (defined as **∆**Cq) between the *EGFR*-mutant Cq and the SPC Cq lies in a predefined range, *a*n automatic report is generated with a “mutation detected” result. Out of this range, a “no mutation detected” result is reported. Time-to-results for the Idylla ctEGFR mutation assay was less than 3 h.

### Data analysis

The clinical performance of the Idylla ctEGFR mutation assay was evaluated based on *EGFR* genotyping for the 38 plasma samples, using Cobas EGFR mutation Test v2 or capture-based NGS approaches as the gold standard.

The sensitivity, specificity and overall agreement of the Idylla ctEGFR mutation test were calculated by considering EGFR-TKI sensitizing mutations as following.

- Sensitivity (Se) = proportion of EGFR-positive samples obtained by Idylla assay among the EGFR-positive samples according to the standard reference method.
- Specificity (Sp) = proportion of EGFR-negative samples obtained by Idylla assay among the EGFR-negative samples according to the standard reference method.
- Overall agreement = number of samples with concordant *EGFR* mutation status between the Idylla assay and the standard reference method out of the overall number of samples.

For all these items, a 95% confidence interval (95% CI) was calculated with exact Clopper-Pearson method.

Agreement between the two conditions of the Idylla assay was investigated by computing a Kappa Value and its 95% CI. A Kappa value greater than 0.6 was considered as good agreement and greater than 0.8 as excellent agreement. Analyses was performed with SAS software version 9.4 (SAS Institute Inc., Cary, NC, USA).

### Limit of detection (LOD) of the Idylla ctEGFR mutation assay

Limits of detection (LOD) of the Idylla system was determined using four different commercial cfDNA solutions that covers 10 *EGFR* variants with predefined allele frequencies of 5%, 1%, 0.1% and 0% (*EGFR* wild-type) respectively (reference HD825, Horizon Discovery Ltd., Cambridge, United Kingdom) (see Supplementary Table S3 online). All DNA solutions (~ 350 ng/tube) were prepared from human cell lines with characterized mutations and fragmented to around 160 bp to mimics cfDNA extracted from human plasma. The cfDNA concentrations of the DNA solutions were determined using the Qubit dsDNA HS assay kit and Qubit 3.0 Fluorometer instrument (ThermoFisher Scientific, Courtaboeuf, France). The number of DNA copies should be approximated considering 300 copies/ng, as previously described^21^. CtDNA/cfDNA samples with different mutant allele frequencies were obtained using 2 mL of plasma collected from healthy donors (reference S4180-500, Dutscher, Brumath, France) spiked with different volumes of DNA solutions (see Supplementary Table S1 online). The LOD was defined for each of the seven mutations detected by the Idylla system as the lowest mutant allele frequency yielding an “*EGFR* mutation detected” result. CfDNA fragment distribution was determined using the DNF-464-0500 High Sensitivity Large Fragment 50 kb Kit (ranging from 1 to 200,000 base pairs (bp)) and the Fragment analyzer instrument (Agilent, Santa Clara, CA, USA) following the supplier’s recommendations (see Supplementary Fig. S1 online). Data were analysed using the PROSize 2.0 2.0.0.51 software. Before its use, the commercial plasma was thawed and centrifuged for 10 min at 6000*g*. Plasma alone was tested negative for *EGFR* mutation by Cobas EGFR mutation assay and cfDNA naturally present in the plasma was quantified as previously described.

## Supplementary Information

Supplementary Figure 1.

Supplementary Information.

## Data Availability

The data generated during the current study are available from the corresponding author on reasonable request.

## References

[1] Bray F (2018). Global cancer statistics 2018: GLOBOCAN estimates of incidence and mortality worldwide for 36 cancers in 185 countries. CA. Cancer J. Clin..

[2] Sher T, Dy GK, Adjei AA (2008). Small cell lung cancer. Mayo Clin. Proc..

[3] Morgensztern D, Ng SH, Gao F, Govindan R (2010). Trends in stage distribution for patients with non-small cell lung cancer: A National Cancer Database survey. J. Thorac. Oncol. Off. Publ. Int. Assoc. Study Lung Cancer.

[4] Lu T (2019). Trends in the incidence, treatment, and survival of patients with lung cancer in the last four decades. Cancer Manag. Res..

[5] Shigematsu H (2005). Clinical and biological features associated with epidermal growth factor receptor gene mutations in lung cancers. J. Natl. Cancer Inst..

[6] Barlesi F (2016). Routine molecular profiling of patients with advanced non-small-cell lung cancer: Results of a 1-year nationwide programme of the French Cooperative Thoracic Intergroup (IFCT). Lancet.

[7] Murray S (2008). Somatic mutations of the tyrosine kinase domain of epidermal growth factor receptor and tyrosine kinase inhibitor response to TKIs in non-small cell lung cancer: An analytical database. J. Thorac. Oncol. Off. Publ. Int. Assoc. Study Lung Cancer.

[8] Sharma SV, Bell DW, Settleman J, Haber DA (2007). Epidermal growth factor receptor mutations in lung cancer. Nat. Rev. Cancer.

[9] Jänne PA, Engelman JA, Johnson BE (2005). Epidermal growth factor receptor mutations in non-small-cell lung cancer: Implications for treatment and tumor biology. J. Clin. Oncol. Off. J. Am. Soc. Clin. Oncol..

[10] Remon J, Steuer CE, Ramalingam SS, Felip E (2018). Osimertinib and other third-generation EGFR TKI in EGFR-mutant NSCLC patients. Ann. Oncol. Off. J. Eur. Soc. Med. Oncol..

[11] Hsu W-H, Yang JC-H, Mok TS, Loong HH (2018). Overview of current systemic management of EGFR-mutant NSCLC. Ann. Oncol. Off. J. Eur. Soc. Med. Oncol..

[12] Yu HA (2013). Analysis of tumor specimens at the time of acquired resistance to EGFR-TKI therapy in 155 patients with EGFR-mutant lung cancers. Clin. Cancer Res. Off. J. Am. Assoc. Cancer Res..

[13] Sequist LV (2011). Genotypic and histological evolution of lung cancers acquiring resistance to EGFR inhibitors. Sci. Transl. Med..

[14] Kobayashi S (2005). EGFR mutation and resistance of non-small-cell lung cancer to gefitinib. N. Engl. J. Med..

[15] Oxnard GR (2011). Acquired resistance to EGFR tyrosine kinase inhibitors in EGFR-mutant lung cancer: Distinct natural history of patients with tumors harboring the T790M mutation. Clin. Cancer Res. Off. J. Am. Assoc. Cancer Res..

[16] Mok TS (2017). Osimertinib or platinum-pemetrexed in EGFR T790M-positive lung cancer. N. Engl. J. Med..

[17] Goldman JW, Noor ZS, Remon J, Besse B, Rosenfeld N (2018). Are liquid biopsies a surrogate for tissue EGFR testing?. Ann. Oncol..

[18] Rolfo C (2018). Liquid biopsy for advanced non-small cell lung cancer (NSCLC): A statement paper from the IASLC. J. Thorac. Oncol..

[19] Francis G, Stein S (2015). Circulating cell-free tumour DNA in the management of cancer. Int. J. Mol. Sci..

[20] Lindeman NI (2018). Updated molecular testing guideline for the selection of lung cancer patients for treatment with targeted tyrosine kinase inhibitors: Guideline from the College of American Pathologists, the International Association for the Study of Lung Cancer, and the Association for Molecular Pathology. J. Mol. Diagn. JMD.

[21] Franczak, C. *et al.* Evaluation of KRAS, NRAS and BRAF mutations detection in plasma using an automated system for patients with metastatic colorectal cancer. *PLoS ONE* **15**, e0227294 (2020). 10.1371/journal.pone.0227294 PMC6961936 31940389

[22] Malapelle U (2017). Profile of the Roche cobas EGFR mutation test v2 for non-small cell lung cancer. Expert Rev. Mol. Diagn..

[23] Kim, Y., Shin, S. & Lee, K.-A. A comparative study for detection of EGFR mutations in plasma cell-free DNA in Korean Clinical Diagnostic Laboratories. *BioMed Res. Int.* **2018**, 7392419 (2018). 10.1155/2018/7392419 PMC5964486 29854785

[24] Guo Q (2019). Detection of plasma EGFR mutations in NSCLC patients with a validated ddPCR lung cfDNA assay. J. Cancer.

[25] Garcia J (2018). Comparison of OncoBEAM and NGS methods to detect plasma EGFR T790M mutations at progression of NSCLC. Ann. Oncol..

[26] Hirotsu Y (2020). Dual-molecular barcode sequencing detects rare variants in tumor and cell free DNA in plasma. Sci. Rep..

[27] De Luca G (2020). Performance of the OncomineTM lung cfDNA assay for liquid biopsy by NGS of NSCLC patients in routine laboratory practice. Appl. Sci..

[28] Delgado-García M (2020). Clinical performance evaluation of the Idylla^TM^ EGFR Mutation Test on formalin-fixed paraffin-embedded tissue of non-small cell lung cancer. BMC Cancer.

[29] Al-Turkmani MR (2020). Rapid EGFR mutation testing in lung cancer tissue samples using a fully automated system and single-use cartridge. Pract. Lab. Med..

[30] Evrard SM (2019). Multicenter evaluation of the fully automated PCR-based Idylla EGFR mutation assay on formalin-fixed, paraffin-embedded tissue of human lung cancer. J. Mol. Diagn. JMD.

[31] Colling R, Bancroft H, Langman G, Soilleux E (2019). Fully automated real-time PCR for EGFR testing in non-small cell lung carcinoma. Virchows Arch. Int. J. Pathol..

[32] Ilie M (2017). Optimization of EGFR mutation detection by the fully-automated qPCR-based Idylla system on tumor tissue from patients with non-small cell lung cancer. Oncotarget.

[33] Lambros L (2017). Evaluation of a fast and fully automated platform to diagnose EGFR and KRAS mutations in formalin-fixed and paraffin-embedded non-small cell lung cancer samples in less than one day. J. Clin. Pathol..

[34] Nunnari, J. *et al.* Rapid EGFR evaluation from used H&E, IHC and FISH diagnostic slides with the Idylla platform. *J. Clin. Pathol.*10.1136/jclinpath-2020-207315 (2021). 10.1136/jclinpath-2020-207315 33597223

[35] Bocciarelli C (2020). Evaluation of the Idylla system to detect the EGFRT790M mutation using extracted DNA. Pathol. Res. Pract..

[36] Grant, J., Stanley, A., Balbi, K., Gerrard, G. & Bennett, P. Performance evaluation of the Biocartis Idylla EGFR Mutation Test using pre-extracted DNA from a cohort of highly characterised mutation positive samples. *J. Clin. Pathol.*10.1136/jclinpath-2020-207338 (2021). 10.1136/jclinpath-2020-207338 33514586

[37] De Luca C (2018). Idylla assay and next generation sequencing: An integrated EGFR mutational testing algorithm. J. Clin. Pathol..

[38] Lee E, Jones V, Topkas E, Harraway J (2021). Reduced sensitivity for EGFR T790M mutations using the Idylla EGFR mutation test. J. Clin. Pathol..

[39] Chevalier L-M (2020). EGFR molecular characterization in non-small cell bronchic cancer: Comparative prospective study by NGS and Idylla platform technologies. Ann. Pathol..

[40] Arcila, M. E. *et al.* Ultrarapid EGFR mutation screening followed by comprehensive next-generation sequencing: A feasible, informative approach for lung carcinoma cytology specimens with a high success rate. *JTO Clin. Res. Rep.* **1**, 100077 (2020). 10.1016/j.jtocrr.2020.100077 PMC7839984 33511359

[41] Momeni-Boroujeni A (2021). Rapid EGFR mutation detection using the Idylla platform: Single-institution experience of 1200 cases analyzed by an in-house developed pipeline and comparison with concurrent next-generation sequencing results. J. Mol. Diagn. JMD.

[42] Boureille A (2020). Rapid detection of EGFR mutations in decalcified lung cancer bone metastasis. J. Bone Oncol..

[43] Torres S (2020). A profile on cobas EGFR Mutation Test v2 as companion diagnostic for first-line treatment of patients with non-small cell lung cancer. Expert Rev. Mol. Diagn..

[44] Marchetti A (2015). Early prediction of response to tyrosine kinase inhibitors by quantification of EGFR mutations in plasma of NSCLC patients. J. Thorac. Oncol..

[45] Gilson P (2020). Evaluation of 3 molecular-based assays for microsatellite instability detection in formalin-fixed tissues of patients with endometrial and colorectal cancers. Sci. Rep..

