# Supplementary figures and images for: Evaluation of the Idylla ctEGFR mutation assay to detect EGFR mutations in plasma from patients with non-small cell lung cancers
Source: Sci Rep. 2021 May 18;11:10470. doi: 10.1038/s41598-021-90091-z (PMC8131701; doi:10.1038/s41598-021-90091-z)

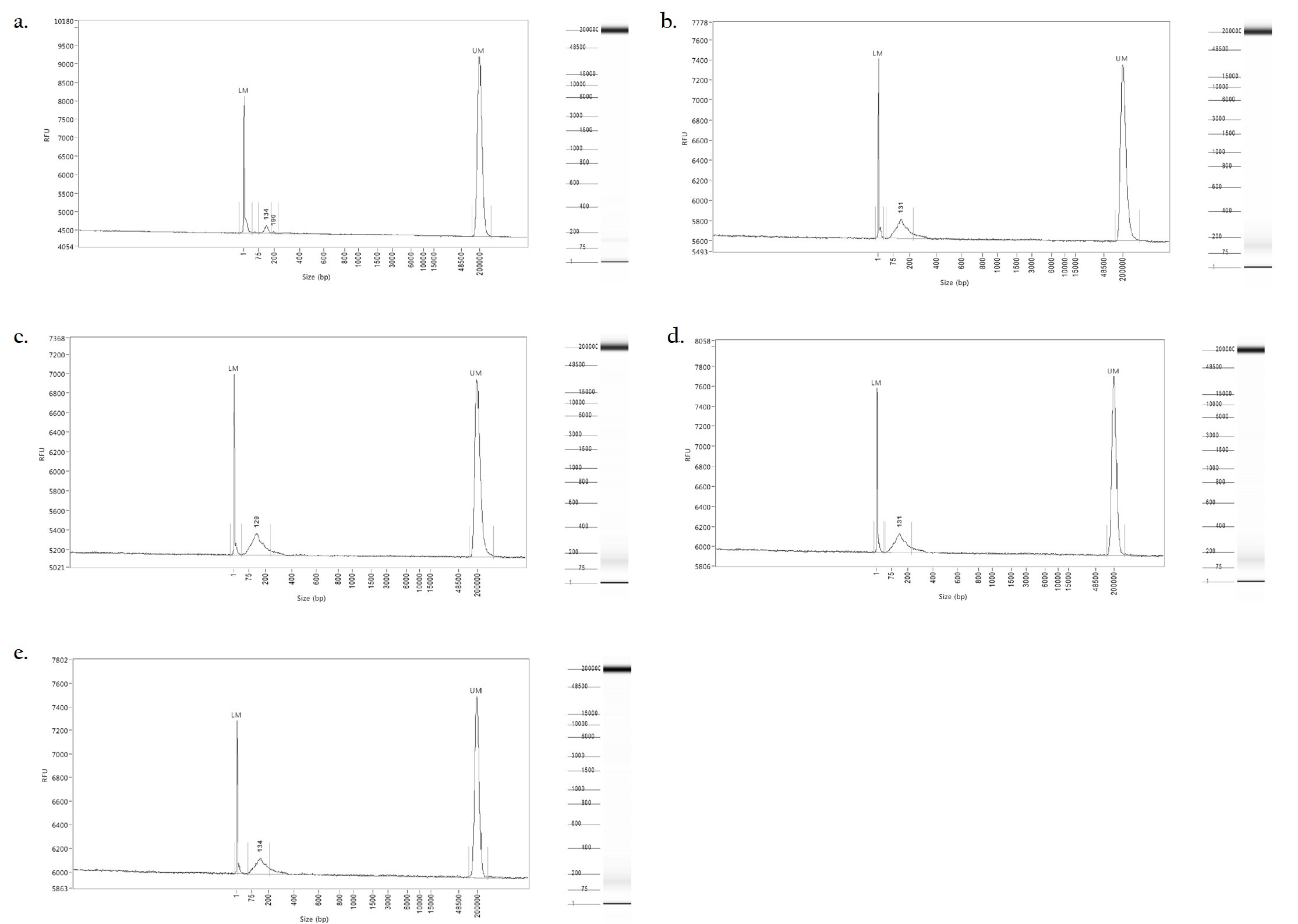

Supplement: Supplementary file 1 — Supplementary Figure 1. [file 41598_2021_90091_MOESM1_ESM.jpg]
