# Supplementary material for: Evaluation of the Idylla ctEGFR mutation assay to detect EGFR mutations in plasma from patients with non-small cell lung cancers
Source: Sci Rep. 2021 May 18;11:10470. doi: 10.1038/s41598-021-90091-z (PMC8131701; doi:10.1038/s41598-021-90091-z)
Supplement: Supplementary file 2 — Supplementary Information. [file 41598_2021_90091_MOESM2_ESM.pdf]

Evaluation of the Idylla ctEGFR mutation assay to detect *EGFR* mutations in plasma from patients with non-small cell lung cancers

Pauline Gilson*, Chloé Saurel, Julia Salleron, Marie Husson, Jessica Demange, Jean-Louis Merlin, Alexandre Harlé

Supplementary information

Supplementary Figure S1. DNA size distribution profiled by fragment analyzer. Data were analysed using the PROSize 2.0 2.0.0.51 software. Length distribution profile of DNA extracted from A) 2 mL of commercial plasma, B) 2 mL of plasma spiked with 0.714% *EGFR*-mutant DNA, C) 2 mL of plasma spiked with 0.144% *EGFR*-mutant DNA, D) 2 mL of plasma spiked with 0.015% *EGFR*-mutant DNA, E) 2 mL of plasma spiked with 0% *EGFR*-mutant DNA.

Size markers correspond to 1 base pair (bp) and 200,000 bp peaks.

Supplementary Table S1. Details on the preparation of artificial plasma samples spiked with *EGFR* multiplex commercial cfDNA solutions. The concentrations of cfDNA naturally present in the commercial plasma were estimated at 0.0485 ng/µL. The cfDNA concentrations of the *EGFR* multiplex 5%, 1%, 0.1% and 0% commercial cfDNA solutions were 16.2 ng/µL, 16.4 ng/µL, 16.9 ng/µL and 14.9 ng/µL respectively.

Sample dilution*		Number of mutant copies (ctDNA)† added	Total number of cfDNA copies ‡	Ratio mutant copies/ total cfDNA copies (%)
Commercial control used	Volume used (µL)			
5% cfDNA solution	1	243.0	34020	0.714%
5% cfDNA solution diluted at 1/4	3	182.3	32805	0.556%
5% cfDNA solution diluted at 3/5	1	145.8	32076	0.455%
1% cfDNA solution	1	49.2	34080	0.144%
1% cfDNA solution diluted at 1/4	3	36.9	32850	0.112%
1% cfDNA solution diluted at 1/5	3	29.5	32112	0.092%
	2	19.7	31128	0.063%
1% cfDNA solution diluted at 1/4	1	12.3	30390	0.041%
1% cfDNA solution diluted at 1/5	1	9.8	30144	0.033%
0.1% cfDNA solution	1	5.1	34215	0.015%
0% cfDNA solution	1	0	33630	0%
/	/	/	29160	Commercial plasma

* Nature and volume (µL) of the commercial cfDNA control added into 2 mL of commercial plasma.

† Number of mutant DNA copies (ctDNA) present in the control and added in the commercial plasma.

‡ Total number of cfDNA copies= number of cfDNA copies in the commercial cfDNA control + number of cfDNAcopies in 2 mL of commercial plasma.

Supplementary Table S2. Details on the *EGFR* mutations covered by the Idylla ctEGFR mutation assay.

Exon	Variant	Nucleotide change	Protein alteration
21	L861Q	c.2582T>A	p.(Leu861Gln)
	L858R	c.2573T>G<br>c.2573_2574delinsGT<br>c.2573_2574delinsGA	p.(Leu858Arg)
20	S768I	c.2303G>T	p.(Ser768Ile)
	T790M	c.2369C>T	p.(Thr790Met)
	Exon 20 insertion	c.2310_2311insGGT	p.(Asp770_Asn771insGly)
		c.2307_2308insGCCAGCGTG	p.(Val769_Asp770insAlaSerVal)
		c.2309_2310delinsCCAGCGTGGAT	p.(Val769_Asp770insAlaSerVal)
		c.2311_2312insGCGTGGACA	p.(Asp770_Asn771insSerValAsp)
		c.2319_2320insCAC	p.(His773_Val774insHis)
19	Exon 19 deletion	c.2238_2248delinsGC<br>c.2239_2248delinsC	p.(Leu747_Ala750delinsPro)
		c.2240_2248del	p.(Leu747_Ala750delinsSer)
		c.2239_2247del	p.(Leu747_Glu749del)
		c.2239_2251delinsC	p.(Leu747_Thr751delinsPro)
		c.2240_2251del	p.(Leu747_Thr751delinsSer)
		c.2235_2249del<br>c.2236_2250del	p.(Glu746_Ala750del)
		c.2239_2253del<br>c.2240_2254del<br>c.2238_2252del	p.(Leu747_Thr751del)
		c.2237_2251del	p.(Glu746_Thr751delinsAla)
		c.2235_2252delinsAAT	p.(Glu746_Thr751delinsIle)
		c.2237_2252delinsT	p.(Glu746_Thr751delinsVal)
		c.2234_2248del	p.(Lys745_Ala750delinsThr)
		c.2236_2253delinsCTA	p.(Glu746_Thr751delinsLeu)
		c.2237_2253delinsTA	p.(Glu746_Thr751delinsVal)
		c.2235_2251delinsAG	p.(Glu746_Thr751delinsAla)
		c.2236_2253delinsCAA	p.(Glu746_Thr751delinsGln)
		c.2230_2249delinsGTCAA	p.(Ile744_Ala750delinsValLys)
		c.2240_2257del	p.(Leu747_Pro753delinsSer)
		c.2237_2255delinsT	p.(Glu746_Ser752delinsVal)

		c.2239_2256del	p.(Leu747_Ser752del)
		c.2236_2253del	p.(Glu746_Thr751del)
		c.2239_2258delinsCA	p.(Leu747_Pro753delinsGln)
		c.2237_2254del	p.(Glu746_Ser752delinsAla)
		c.2238_2255del	p.(Glu746_Ser752delinsAsp)
		c.2236_2256delinsATC	p.(Glu746_Ser752delinsIle)
		c.2237_2256delinsTT<br>c.2237_2256delinsTC<br>c.2235_2255delinsGGT	p.(Glu746_Ser752delinsVal)
		c.2238_2258del	p.(Leu747_Pro753del)
		c.2236_2256del	p.(Glu746_Ser752del)
		c.2253_2276del	p.(Ser752_Ile759del)
18	G719A/C/S	c.2156G>C	p.(Gly719Ala)
		c.2155G>T<br>c.2154_2155delinsTT	p.(Gly719Cys)
		c.2155G>A	p.(Gly719Ser)

Supplementary Table S3. Details on the *EGFR* mutations covered by the commercial cfDNA solutions (reference HD825, Horizon Discovery).

gene	exon	variant	Nucleotide change	Protein alteration	Mutations detected by the Idylla ctEGFR mutation assay
EGFR	21	L861Q	c.2582T>A	p.(Leu861Gln)	X
		L858R	c.2573T>G	p.(Leu858Arg)	X
	20	S768I	c.2303G>T	p.(Ser768Ile)	X
		V769_D770ins	c.2307_2308insGCCAGCGTG	p.(Val769_Asp770ins)	X
		T790M	c.2369C>T	p.(Thr790Met)	X
		C797S	c.2389T>A	p.(Cys797Ser)	
	19	E746_A750del	c.2236_2250del15	p.(Glu746_Ala750del)	X
	18	G719S	c.2155G>A	p.(Gly719Ser)	X
	12	S464L	c.1391C>T	p.(Ser464Leu)	
		G465R	c.1393G>A	p.(Gly465Arg)	
